# Analysis of the Content of Chromium in Certain Parts of the Human Knee Joint

**DOI:** 10.3390/ijerph15051013

**Published:** 2018-05-17

**Authors:** Wojciech Roczniak, Barbara Brodziak-Dopierała, Elżbieta Cipora, Agata Jakóbik-Kolon, Magdalena Konieczny, Magdalena Babuśka-Roczniak

**Affiliations:** 1 The Jan Grodek Higher Vocational State School, Medical Institute, 21 Mickiewicza Str., 38-500 Sanok, Poland; elacipora@interia.pl (E.C.); boras86@wp.pl (M.K.); magda_babuska@vp.pl (M.B.-R.); 2 Department of Toxicology and Bioanalysis, School of Pharmacy with the Division of Laboratory Medicine, Medical University of Silesia, 4 Jagiellonska Str., 41-200 Sosnowiec, Poland; bbrodziak@sum.edu.pl; 3 Department of Inorganic, Analytical Chemistry and Electrochemistry, Faculty of Chemistry, Silesian University of Technology, 6 B. Krzywoustego Str., 44-100 Gliwice, Poland; agata.jakobik-kolon@polsl.pl

**Keywords:** knee joint tissues, chromium

## Abstract

Chromium is an essential microelement in the human body. It exerts an effect on bones by modulating their biochemical parameters: alkaline phosphatase (ALP) and tartrate-resistant acid phosphatase (TRAP). With considerable accumulation of chromium in the skeleton, the activity of alkaline phosphatase was found to decrease, which affected bone formation rate. The study objective was to analyze chromium content in the knee tissues. Tissues for analysis were obtained during endoprosthesoplasty of the knee joint and included tibia, femur, and meniscus tissues. Samples were collected from 50 patients, including 36 women and 14 men. The analysis was performed using the inductively coupled plasma atomic emission spectroscopy (ICP-AES) method, by means of a Varian 710-ES apparatus. The results revealed no significant differences in the content of chromium in the knee joint tissues between women and men. The highest level of chromium was found in the femoral bone of the knee joint, then in the meniscus, and was lowest in the tibia, although the differences were statistically insignificant. Chromium content increased with age.

## 1. Introduction

Chromium in living organisms occurs as a trace element, yet its presence is extremely important. It coordinates the normal function of the body through proper metabolic transformations. The dietary sources of chromium include seafood, fish, or whole-meal cereal products [1,2]. The optimum level of chromium in the body allows for maintaining proper sugar level in the blood, normalizes cholesterol level, takes part in the metabolism of fats, proteins and carbohydrates, and reduces appetite [3]. Research into osteoblasts revealed that chromium inhibits the level of osteocalcin, which when high, may accompany osteoporosis. It does not block collagen production [4]. Reduced bone resorption was observed in women after menopause, and thus adequate chromium supplementation was found to prevent osteoporosis [5].

Toxic chromium compounds are absorbed via inhalation, through the skin, and poorly, by the alimentary tract. Chromium compounds absorbed through the respiratory system are more toxic than when obtained through the digestive system. They act on skin and mucosa as a strong allergen, and inhibit many enzymes, causing damage to the respiratory and digestive systems. They also have carcinogenic, mutagenic, embryogenic, and teratogenic effects [6].

Due to its properties, chromium is used in the production of orthopedic implants. There is still some concern regarding the local toxicity of chromium contained in prostheses and a number of studies have been conducted on the issue [1,7,8].

Molecular metals released from the wear of articular implants include mainly colloidal alloys of Cr-Co and some oxides (Cr_2_O_3_ and CoO). Light microscopy of periprosthetic tissues revealed that metal molecules are easily phagocytized by macrophages present in tissues and transported in the endosomal-lysosomal compartment. In endosomes, due to the acidic pH, metals get transformed into ions with varied degrees of oxidation [1,9,10,11,12].

Proteomic analysis indicates that Cr (III) ions can bind several cell proteins, including enzymes involved in redox reactions (catalase, superoxide dismutase, and glutathione peroxidase) in metabolism (arginase 1 and transglutaminase 3), in molecular transport (transferrin and hemoglobin), in cell migration (Annexin A1 and Annexin A2), and in cell signaling (leukotriene hydrolase A4 and phosphodiesterase 3A). A few cell pathways and functions can potentially be at risk via metal binding. Cr ions can displace other metal ions present in metalloproteins and affect their activity [1].

There is a strong positive correlation of the number of Cr and Co ions with tissue and oxidative damage. Patients with the highest quantity of Cr and Co ions show the highest levels of protein carbonylation, a well-known consequence of oxidative stress. The effects of oxidative stress are associated with tissue degeneration and necrosis [1,13].

The aim of the study was to determine chromium content in chosen components of the knee joint: the tibia, the femur, and the meniscus. The effect of smoking on the level of chromium was investigated.

## 2. Material and Methods

The study material included parts of the knee joint obtained during endoprosthesoplasty in the dr Janusz Daab Hospital of Trauma Surgery in Piekary Śląskie. Biological samples were obtained from patients living in Silesia Province. Samples were collected from 50 patients—36 women and 14 men. In 26 patients the right leg, and in 24 patients the left leg, were involved. The mean age of the whole study population was 67.5 years, being slightly lower in women—67.2 years than in men—68.1 years. In the study group, patients complained of pain of 10 years’ duration. A detailed description of the test group patients is shown in Table 1.

The study was approved by the Bioethics Committee No 2/2013 of 18 June 2013. Degenerative disease of the knee joint and considerable pain were indications for this type of procedure. Surgeries were performed in subarachnoid anesthesia, with patients in the prone position. An Esmarch bandage was used for exsanguination of the limb. The frontal surface of the knee joint was exposed following standard preparation of the operation field (applying antiseptic and aseptic techniques) with straight midline incision. The joint was opened at the medial side and the hypertrophic synovium was removed. Using ZIMMER instrumentation (Zimmer Biomed, Warsaw, IN, USA), the femoral part of the knee joint was prepared, by preparing the distal femur and performing femoral epicondyle osteotomy. Next, damaged menisci were removed, and by using ZIMMER instrumentation the tibial part was prepared (resection of the tibial plateau). In this way, the osseous components, cartilages, and parts of menisci were used for measurements.

The material samples were described and stored in modified polyethylene containers, in a freezer at a temperature of −22 °C.

Tissue samples with a known mass were mineralized using 4 cm³ of spectrally pure HNO_3_ (V) (Supra pure, Merck, Dormstadt, Germany), in a microwave mineralizer Magnum II (Ertec, Wrocław, Poland). The samples were placed one by one in a Teflon vessel and were added mineralization. Mineralization was a two-stage procedure. The first stage lasted 2 min. at 20 bar max. pressure and 255 °C max. temperature, whereas the second stage was of 6 min. duration at 45 bar max pressure and 285 °C max. temperature. The post-mineralization solution was transferred to a 25 cm^3^ flask and then diluted to the ml mark with redistilled water.

The content of calcium, phosphorus, sodium, and magnesium in mineralized samples was determined using inductively coupled plasma atomic emission spectrometry (ICP-AES). A Varian 710-ES spectrometer (Palo Alto, CA, USA) equipped with a OneNeb nebulizer was utilized. The following parameters were used: RF (radio frequency) power 1.0 kW, plasma flow 15 L/min, auxiliary flow 1.5 L/min, nebulizer pressure 210 kPa, pump rate 15 rpm, and emission lines of Cr: λ = 267.716 nm. The calibration curve method was applied. The standard solutions of 1 mg/mL (Millipore SAS, Molsheim, France) as well as deionized water (Elix Essential 10, Merck Millipore, Burlington, MA, USA) were used. The results are the average of the concentrations obtained for all analytical lines used for the element, with standard deviations not exceeding 1.5%. The accuracy of the analysis was controlled using Standard Reference Material 1400 Bone Ash (NIST—National Institute of Standards and Technology, Monsanto Co., St. Louis, MO, USA).

## 3. Results

Since chromium content in the knee joint tissue did not show normal distribution, nonparametric tests were used to calculate the differences. The U Mann–Whitney test was applied for two samples and the Kruskal–Wallis ANOVA rank test was applied for many samples. The level of significance at *p* ≤ 0.05 was considered statistically significant.

No statistically significant differences were found in chromium content in the respective components of the knee joint. The level of chromium was the highest in the femur (1.64 μg/g), then in the tibia (1.27 μg/g), and the lowest in the meniscus—1.18 μg/g. Chromium content in the tissues of the knee joint was slightly higher in men (1.38 μg/g) as compared to women (1.36 μg/g), the difference being statistically insignificant. Chromium content in the respective components of the knee joint was the highest in the femur both in men and women. In women, the level of chromium was the lowest in the meniscus, whereas in men in the tibia (Table 2).

**Figure 1:**
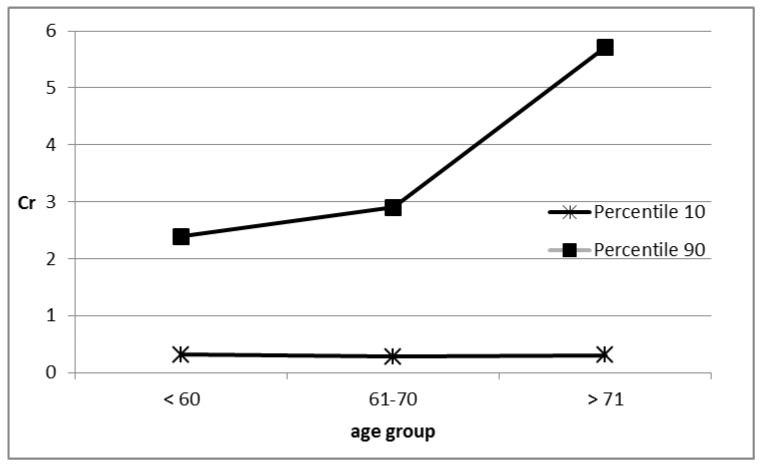
The chromium content changes in age groups.

The Kruskal–Wallis ANOVA rank test showed significant age-dependent differences in the content of chromium at *p* = 0.03. It was the lowest in the age group of 60 (0.88 μg/g), increasing with age to 1.08 μg/g (the 61–70-year-old group) and to 1.78 μg/g above the age of 70. Figure 1 shows the changes in chromium content (percentile 10 and 90) in age groups.

**Figure 2:**
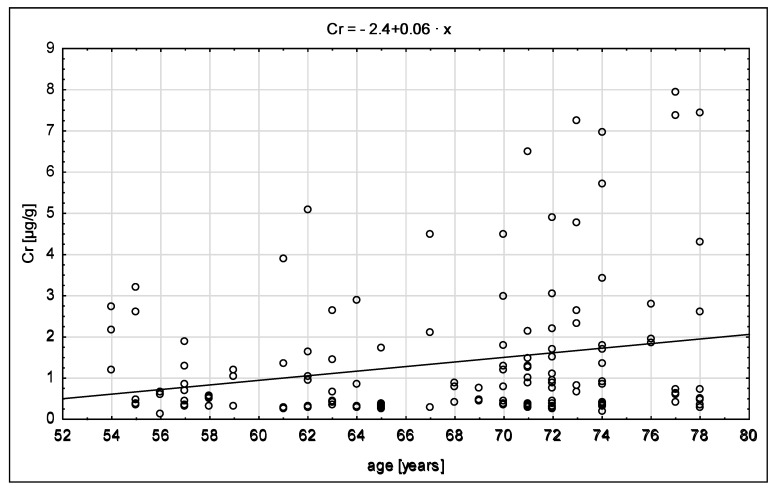
The distribution of chromium content in relation to age.

Figure 2 presents the scatter diagram of chromium content depending on age. The level of chromium increases with age, which is also described using the regression equation Cr = 0.06x − 2.40. The value of the β coefficient is close to zero, which is interpreted as a weak correlation.

The analysis of chromium content depending on the place of living revealed no statistically significant differences. The level of chromium was the highest in patients from towns of up to 20,000 population (2.30 μg/g), as compared to the rural areas (1.20 μg/g).

Tobacco smoking increases the content of some metals in the body resulting from the presence of these components in tobacco smoke. This usually refers to cadmium, due to high accumulation of this element in the tobacco plant. We failed to observe any significant differences between smokers and non-smokers. The content of chromium was lower in the group of smokers as compared to those who do not smoke (1.00 vs. 1.47 μg/g).

Occupational exposure can cause an increase in the level of elements in human tissues. In the study population, subjects with occupational exposure accounted for only 10%. No significant differences were observed between people exposed to occupational hazards and those who were not (1.25 vs. 1.38 μg/g, respectively).

## 4. Discussion

Due to its involvement in glucose metabolism, chromium belongs to microelements consumed by an increasing number of people. The skeleton is a target for most metals, leading to their bioaccumulation or acting as a storage site for macro- and microelement homeostasis [14]. Bones, due to their structure, long regeneration time, and accumulation potential, can be used as a biomarker of exposure to heavy metals. Elements in bones can accumulate for several years. The use of human tissues for investigations is quite frequent [15,16,17,18,19,20,21,22].

The current study revealed that the level of chromium accumulated in the bone tissue was similar to its content in the connective tissue. Thus, chromium accumulation is opposite to that of cadmium [23,24]. In the study concerning the content of chromium in the hip joint, its level in the spongy bone was approximately 3 times higher compared to other parts of this joint.

In patients with degenerative lesions in both knees, the level of chromium was higher (1.40 µg/g) compared to those with degenerative lesions in one knee (1.29 µg/g). However, the differences were not statistically significant. Kuo et al. [15] showed that in the case of various hip joint complaints the content of chromium was lowest in the group of patients with fractures (4.96 μg/g) and the highest in those with other ailments (21.33 μg/g).

In bones obtained from excavations, the content of chromium ranged between 19 and 28 μg/g in the respective historical epochs [20]. These values are substantially higher compared to the results obtained in our study. This might have been caused by the conditions, place of burial, and type of soil where the bones were found.

The content of chromium in the bones of the hip joint in a population from an area of highly industrialized Taiwan was 11.9 μg/g [15], being a eight times higher as compared to the value for the knee joint (1.36 μg/g).

Taking into account the effect of age on the content of chromium, there is an age-dependent increase from 0.88 to 1.78 μg/g. Similarly, in the hip joint tissues, the level of chromium was 3.14 μg/g in the youngest age group and 11.64 μg/g in the oldest over 80-year-old group. Kuo et al. [15] found the lowest level of chromium in the bones of Taiwan inhabitants aged up to 40 (5.36), whereas the highest in the 41–60-year-old (21.68).

In the population of the Tarragona region in Spain, Garcia et al. [21] determined the level of chromium at 0.33 μg/g (range of changes 0.2–5.8), which is higher compared to our study. Garcia confirmed the lack of statistically significant differences in the level of chromium in the bone tissue in different age groups. The level was higher in men and among smokers. We found no statistically significant differences in the content of chromium between women and men, and between smokers and non-smokers. 

In the ribs of the Japanese, the level of chromium determined by Yoshinaga et al. [22] was much higher (6 μg/g).

In our study on the hip joint, the level of chromium was a few times higher (7.53 μg/g) as compared to the knee joint (1.36 μg/g) [25]. These differences may be due to the different methodologies used for analysis. Our results were higher than those obtained in Poland for the hip joint. The content of chromium determined by Lanocha-Arendarczyk et al. [17] was 0.45 g/kg in the articular cartilage and 0.66 g/kg in the cortical bone and spongy bone. On the other hand, the content of chromium in the knee joint was almost identical as in the femoral head (1.36 vs. 1.33 μg/g, respectively), as reported from Poland by Zioła-Frankowska et al. [18].

## 5. Conclusions

The highest chromium content was found in the femoral bone, then in the tibia, and was lowest in the meniscus; however, the differences were not statistically significant in the respective parts of the knee joint.

Sex was not the differentiating factor for chromium content in the knee joint tissues. Chromium content in the tissues examined increased with age.

The effects of place of residence or occupational hazards on chromium content in the tissues of the knee joint were not confirmed.

## Figures and Tables

**Table 1:** Information about the study group patients (AM—arithmetic mean; SD—standard deviation).

Parameters	Whole Population *n* = 50	Females *n* = 36	Males *n* = 14
Age (years)			
AM ± SD	67.46 ± 7.11	67.22 ± 7.09	68.07 ± 7.20
range	54–78	54–78	56–78
Body weight (kg)			
AM ± SD	83.54 ± 14.56	81.45 ± 14.19	88.58 ± 14.56
range	54–115	54–115	66–108
Height (cm)			
AM ± SD	164.37 ± 9.32	160.24 ± 6.14	174.33 ± 8.11
range	149–189	149–173	165–189
Smokers (*n*, %)			
- nonsmokers	20 (40%)	19 (38%)	1 (2%)
- smokers	21 (42%)	10 (20%)	11 (22%)
- smokers in the past	9 (18%)	5 (10%)	4 (8%)
Place of residence (%)			
Village	11 (22%)	7 (14%)	4 (8%)
Town	39 (78%)	29 (58%)	10 (20%)
Knee (%)			
Left	24 (48%)	18 (36%)	6 (12%)
Right	26 (52%)	18 (36%)	8 (16%)
Beginning pain (years, %)			
< 5	16 (32%)	11 (22%)	5 (10%)
< 10	21 (42%)	15 (30%)	6 (12%)
10	13 (26%)	10 (20%)	3 (9%)
Earlier knee endoprosthesis (%)			
Yes	13 (26%)	10 (20%)	3 (6%)
No	37 (74%)	26 (52%)	11 (22%)
Degenerative changes in the other knee (%)			
Yes	33 (66%)	23 (46%)	10 (20%)
No	17 (34%)	13 (26%)	4 (8%)
Contact with chemicals in the workplace (factory polyvinyl chloride, zinc smelter) (%)	3 (6%)	1 (2%)	2 (4%)

**Table 2:** Statistical characteristics for concentration of chromium in tissues of the knee joint (mg/kg) (AM—arithmetic mean; SD—standard deviation; Med—median; CV—coefficient variability).

	AM ± SD	Med	Range	Percentile 10	Percentile 90	CV
MEN						
Meniscus	1.33 ± 1.11	0.80	0.13–3.00	0.34	2.90	84
Tibia	1.25 ± 1.65	0.53	0.28–6.49	0.30	2.21	133
Femur	1.57 ± 1.67	0.90	0.30–5.72	0.32	4.49	106
WOMEN						
Meniscus	1.12 ± 1.38	0.65	0.28–6.97	0.32	2.09	123
Tibia	1.28 ± 1.56	0.67	0.25–7.25	0.30	3.89	122
Femur	1.67 ± 2.17	0.61	0.20–7.95	0.27	4.90	130
TOTAL						
Meniscus	1.18 ± 1.30	0.67	0.13–6.97	0.32	2.84	110
Tibia	1.27 ± 1.57	0.60	0.25–7.25	0.30	3.66	124
Femur	1.64 ± 2.03	0.76	0.20–7.95	0.29	4.84	124

## References

[1] Scharf B., Clement C.C., Zolla V., Perino G., Yan B., Elci S.G., Purdue E., Goldring S., Macaluso F., Cobelli N. (2014). Molecular analysis of chromium and cobalt-related toxicity. Sci. Rep..

[2] Granadino V.A., Parra de Machado L., Romero R.A. (1994). Determination of total chromium in whole blood, blood components, bone, and urine by fast furnace program electrothermal atomization AAS and using neither analyte isoformation nor background correction. Anal. Chem..

[3] Kabata-Pendias A., Mukherjee A.B. (2007). Trace Elements from Soil to Human.

[4] Allen M.J., Myer B.J., Millett P.J., Rushton N. (1997). The effects of particulate cobalt, chromium and cobalt-chromium alloy on human osteoblast-like cells in vitro. J. Bone Jt. Surg. Br..

[5] McCarty M.F. (1995). Anabolic effects of insulin on bone suggest a role for chromium picolinate in preservation of bone density. Med. Hypotheses.

[6] Flores-Cano J.V., Leyva-Ramos R., Carrasco-Marin F., Aragón-Piña A., Salazar-Rabago J.J., Leyva-Ramos S. (2016). Adsorption mechanism of chromium (III) from water solution on bone char: Effect of operating conditions. Adsorption.

[7] Wang J.Y., Wicklund B.H., Gustilo R.B., Tsukayama D.T. (1996). Titanium, chromium and cobalt ions modulate the release of bone-associated cytokines by human monocytes/macrophages in vitro. Biomaterials.

[8] Rakow A., Schoon J., Dienelt A., John T., Textor M., Duda G., Perka C., Schulze F., Ode A. (2016). Influence of particulate and dissociated metal-on-metal hip endoprosthesis wear on mesenchymal stromal cells in vivo and in vitro. Biomaterials.

[9] Davda K., Lali F.V., Sampson B., Skinner J.A., Hart A.J. (2011). An analysis of metal ion levels in the joint fluid of symptomatic patients with metal-on-metal hip replacements. J. Bone Jt. Surg. Br..

[10] Dobbs H.S., Minski M.J. (1980). Metal ion release after total hip replacement. Biomaterials.

[11] Luetzner J., Krummenauer F., Lengel A.M., Ziegler J., Witzleb W.C. (2007). Serum metal ion exposure after total knee arthroplasty. Clin. Orthop. Relat. Res..

[12] McPhee I.B., Swanson C.E. (2007). Metal ion levels in patients with stainless steel spinal instrumentation. Spine.

[13] Barceloux D.G. (1999). Chromium. J. Toxicol. Clin. Toxicol..

[14] Vidaud C., Bourgeois D., Meyer D. (2012). Bone as target organ for metals: The case of f-elements. Chem. Res. Toxicol..

[15] Kuo H.W., Kuo S.M., Chou C.H., Lee T. (2000). Determination of 14 elements in Taiwanese bones. Sci. Total Environ..

[16] Brodziak-Dopierała B., Kwapuliński J., Sobczyk K., Wiechuła D. (2013). Distribution of magnesium, calcium, sodium and potassium in tissues of the hip joint. Magnes. Res..

[17] Lanocha-Arendarczyk N., Kosik-Bogacka D.I., Kalisinska E., Sokolowski S., Kolodziej L., Budis H., Safranow K., Kot K., Ciosek Z., Tomska N. (2016). Influence of environmental factors and relationships between vanadium, chromium, and calcium in human bone. Biomed. Res. Int..

[18] Zioła-Frankowska A., Kubaszewski Ł., Dąbrowski M., Kowalski A., Rogala P., Strzyżewski W., Łabędź W., Uklejewski R., Novotny K., Kanicky V. (2015). The content of the 14 metals in cancellous and cortical bone of the hip joint affected by osteoarthritis. Biomed. Res. Int..

[19] Brodziak-Dopierała B., Kwapuliński J., Kusz D., Gajda Z., Sobczyk K. (2009). Interactions between concentrations of chemical elements in human femoral heads. Arch. Environ. Contam. Toxicol..

[20] Kosugi H., Hanihara K., Suzuki T., Himeno S.I., Kawabe T., Hongo T., Morita M. (1986). Elemental composition of ancient Japanese bones. Sci. Total Environ..

[21] Garcia F., Ortega A., Domingo J.L., Corbella J. (2001). Accumulation of metals in autopsy tissues of subjects living in Tarragona county, Spain. J. Environ. Sci. Health.

[22] Yoshinaga J., Suzuki T., Morita M., Hayakawa M. (1995). Trace elements in ribs of elderly people and elemental variation in the presence of chronic diseases. Sci. Total Environ..

[23] Roczniak W., Brodziak-Dopierała B., Cipora E., Jakóbik-Kolon A., Kluczka J., Babuśka-Roczniak M. (2017). Factors that affect the content of cadmium, nickel, copper and zinc in tissues of the knee joint. Biol. Trace Elem. Res..

[24] Roczniak W., Brodziak-Dopierała B., Cipora E., Mitko K., Jakóbik-Kolon A., Konieczny M., Babuśka-Roczniak M. (2017). The content of structural and trace elements in the knee joint tissues. Int. J. Environ. Res. Public Health.

[25] Brodziak-Dopierała B., Kwapuliński J., Sobczyk K., Wiechuła D. (2015). Chromium content in the human hip joint tissues. Biomed. Environ. Sci..

